# Multi-millijoule terahertz emission from laser-wakefield-accelerated electrons

**DOI:** 10.1038/s41377-022-01068-0

**Published:** 2023-02-06

**Authors:** Taegyu Pak, Mohammad Rezaei-Pandari, Sang Beom Kim, Geonwoo Lee, Dae Hee Wi, Calin Ioan Hojbota, Mohammad Mirzaie, Hyeongmun Kim, Jae Hee Sung, Seong Ku Lee, Chul Kang, Ki-Yong Kim

**Affiliations:** 1 Center for Relativistic Laser Science, Institute for Basic Science, Gwangju, 61005 Korea; 2 Department of Physics and Photon Science, Gwangju Institute of Science and Technology (GIST), Gwangju, 61005 Korea; 3 Laser and Plasma Research Institute, Shahid Beheshti University, Tehran, Iran; 4 Advanced Photonics Research Institute, GIST, Gwangju, 61005 Korea; 5 Institute for Research in Electronics and Applied Physics and Department of Physics, University of Maryland, College Park, Maryland 20742 USA

**Keywords:** Terahertz optics, Plasma-based accelerators

## Abstract

High-power terahertz radiation was observed to be emitted from a gas jet irradiated by 100-terawatt-class laser pulses in the laser-wakefield acceleration of electrons. The emitted terahertz radiation was characterized in terms of its spectrum, polarization, and energy dependence on the accompanying electron bunch energy and charge under various gas target conditions. With a nitrogen target, more than 4 mJ of energy was produced at <10 THz with a laser-to-terahertz conversion efficiency of ~0.15%. Such strong terahertz radiation is hypothesized to be produced from plasma electrons accelerated by the ponderomotive force of the laser and the plasma wakefields on the time scale of the laser pulse duration and plasma period. This model is examined with analytic calculations and particle-in-cell simulations to better understand the generation mechanism of high-energy terahertz radiation in laser-wakefield acceleration.

## Abstract (web summary)

The electrons, accelerated by the laser ponderomotive force and subsequent plasma wakefields, radiate broadband emission continuously along the laser propagation direction, resulting in multi-mJ THz radiation in the far field.

## Graphical abstract

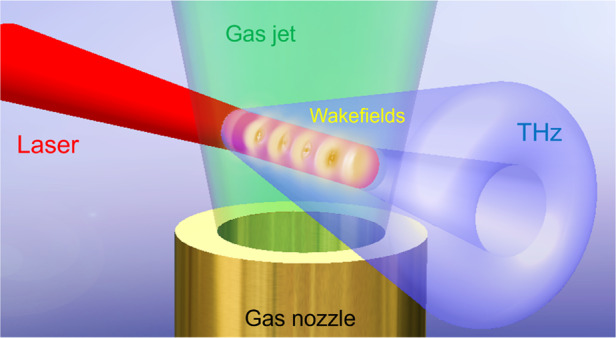

## Introduction

The terahertz (THz) gap, a frequency band lying between the microwave and infrared regions of the electromagnetic spectrum where conventional technologies are inefficient in generating and detecting the radiation, is being rapidly closed by development of new THz sources and detectors^1–3^. In particular, laser-based THz sources are of great interest due to their capability of producing coherent, single-cycle-to-multicycle, broadband (or narrowband) radiation. Such sources can also provide natural synchronization with the driving laser, allowing ultrafast time-resolved spectroscopy and imaging^3^. Recently, high-power femtosecond lasers have been used to produce strong THz radiation, as well as to explore novel THz-driven phenomena such as molecular alignment^4^, harmonic generation^5^, and electron acceleration^6^.

Among many laser-based sources, laser-plasma-based ones are well suited for high-power THz generation. Plasmas are already ionized and thus can sustain high electromagnetic fields, with little or no concern about material damage when high-power laser pulses are focused into a small volume for energy-scalable THz generation. Since the pioneering work by Hamster et al.^7,8^ coherent THz generation from laser-produced gaseous and solid-density plasmas has been extensively investigated^9–20^. In gases, single- or two-color laser-produced plasmas can generate coherent broadband THz radiation^9–15^ by ultrafast laser-driven currents^12,13^. In two-color laser mixing, the laser-to-THz conversion efficiency increased up to the percent level by using mid-infrared laser drivers^14,15^. High-energy THz radiation was also observed from laser-irradiated, high-density plasma targets based on liquids^16,17^ and solids^18–20^. Recently, tens of mJ of THz energy was observed from a metal foil irradiated by high-energy (~60 J) picosecond laser pulses^20^. Unlike gas targets, high-density ones, however, often pose target debris and target reloading issues, which makes them unfavorable for use in continuous or high-repetition-rate (>kHz) operation.

Laser-wakefield acceleration (LWFA), a gaseous plasma-based compact electron accelerator scheme^21^, is another source of broadband electromagnetic radiation^22^. A relativistic electron bunch produced in LWFA can emit THz radiation when it exits the plasma-vacuum boundary by coherent transition radiation (CTR)^23–25^. This occurs when the bunch size becomes compared to or less than the wavelength of the emitted THz radiation, and the THz fields produced by individual electrons coherently add up in the radiation direction. In this case, the emitted THz energy scales with the square of the bunch charge. Experimentally, <100 nJ of THz energy was observed from LWFA with a 10-TW-class laser^26^, and the waveforms of THz radiation were measured in single-shots and also utilized to diagnose the electron bunches themselves^26,27^. Since then, however, almost no experiment has been reported regarding THz emission from LWFA, in particular high-energy THz generation with more powerful laser drivers. Until now the output THz energy from LWFA has not exceeded the microjoule (μJ) level, and no THz energy scaling has been studied.

In this paper, we present significantly enhanced THz generation, at the multi-mJ level, in LWFA by using a 150-TW laser at the Center for Relativistic Laser Science (CoReLS)^28^. We have examined LWFA and THz generation under various target conditions and simultaneously characterized both beams to have a better understanding of the origin of THz generation in LWFA. Our experimental results suggest that multi-mJ THz generation is not solely explained by the CTR model. We examine another possible mechanism for THz generation in LWFA, which is coherent radiation from plasma electrons accelerated by the laser ponderomotive force and plasma wakefields over the period of the laser pulse duration, which typically covers tens of THz frequencies.

## Results

### Experimental method

**Fig. 1:**
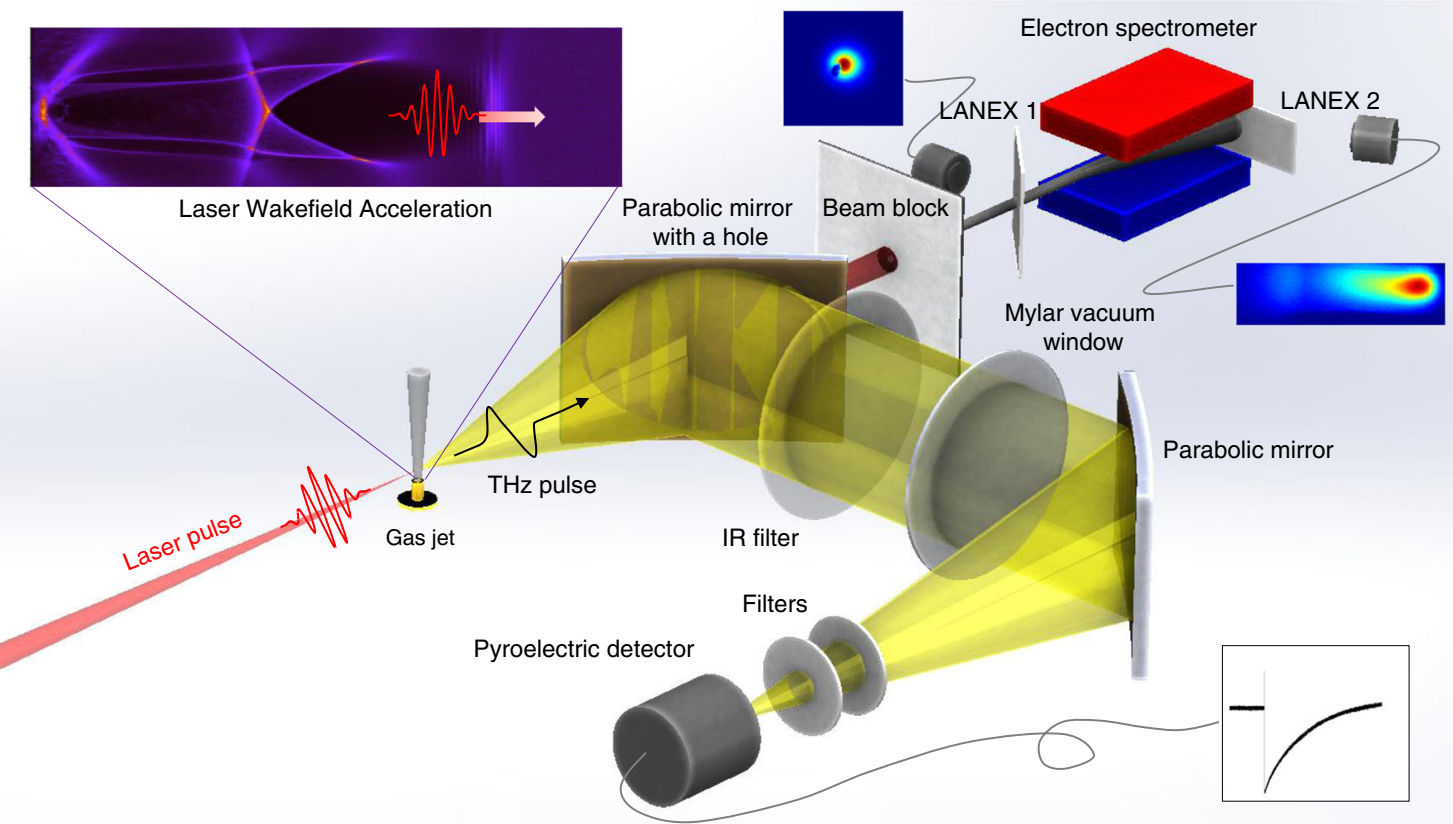
Schematic of laser-driven electron acceleration and THz generation. The laser pulse ionizes a gas jet and accelerates plasma electrons via LWFA, simultaneously generating THz radiation. The electron beam diagnostics include scintillating screens—Lanex 1 for beam profiling and Lanex 2 for measuring the electron energy spectrum after the beam passes through an electron spectrometer with a 1-T dipole magnet. THz radiation emitted from the plasma is collimated, transported outside the vacuum chamber, and then refocused onto a pyroelectric detector for detection

A schematic of our experiment is shown in Fig. 1. Laser pulses of 800 nm, >27 fs, and <2.7 J with linear (horizontal) polarization were focused into a gas jet by a concave mirror with the focal length of 1.5 m. After optimized with adaptive optics, the focal spot size was 22 μm in the full width at half maximum (FWHM), which provided the peak laser intensity of 5.2 × 10^18 ^W/cm^2^ at the focus. The corresponding normalized vector potential was $a_0 = eA/m_ec^2$ = 1.6, where *e* is the electron charge, *A* is the laser vector potential, *m*_*e*_ is the electron mass, and *c* is the speed of light. The laser intensity was high enough to ionize the gas and accelerate the liberated electrons via LWFA. After the interaction, the laser and electron beams propagated through a hole of a 90° off-axis parabolic (OAP) gold mirror. The laser beam was then blocked by aluminum foils (1-mm thickness overall) while the electron beam propagated through the foils until detected by a scintillating screen (Lanex 1) and an electron spectrometer consisting of a dipole magnet and Lanex 2. See “Materials and methods” section for more details on the setup and laser/electron beam diagnostics.

The THz radiation produced from the plasma was collected by the same holed (12-mm diameter) OAP mirror (50-mm diameter, 102-mm focal length), providing a half-collection angle of 3.4°−14°. The collected THz radiation was directed outside the vacuum chamber through a 180-μm-thick mylar window and then focused by another OAP mirror (60-mm clear-aperture diameter, 230-mm focal length) onto a pyroelectric detector for the measurement of energy. From source to detector, several filters were placed in the THz beam path, including infrared (IR) filters and a lowpass filter providing a cut-off frequency of 23.1 THz. More details on the pyroelectric detector and filters are provided in “Materials and methods” section.

### Electron and THz beam correlation

**Fig. 2:**
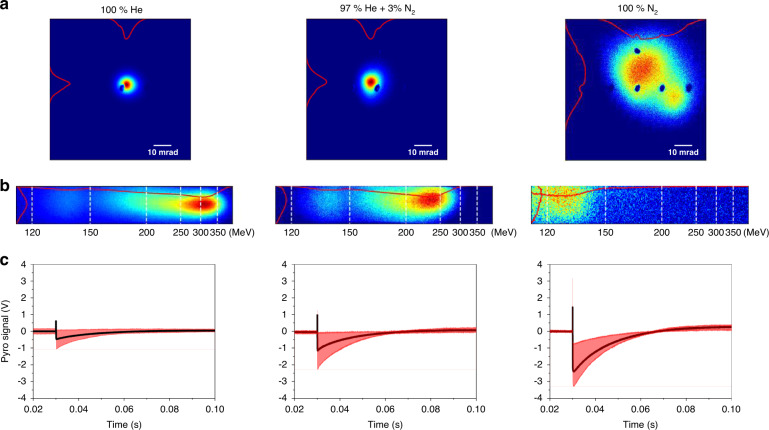
Sample data taken with three different gas species—100% helium, 97% helium mixed with 3% nitrogen, and 100% nitrogen. **a** Electron beam profiles imaged by Lanex 1. **b** Electron energy spectra taken with Lanex 2. **c** Pyroelectric signals taken with an oscilloscope, with the black and red lines representing the averaged and minimum/maximum traces, respectively. In (**a**), the dark dots are coordinate points pen-marked on Lanex 1

Figure 2 shows typical electron beam profiles, energy spectra, and THz signals obtained with three types of gas species—100% helium (He), 97% He mixed with 3% nitrogen (N_2_), and 100% N_2_. In the case of pure helium, the electron beam was accelerated in the bubble (or blow-out) regime with self-injection^21^. The electron beam was well confined within a divergence angle of ~5 mrad, and the energy exceeded ~300 MeV with charge of ~5 pC. In the mixed gas case, 3% nitrogen was added to induce ionization injection for stable electron beam generation^29–31^. This made the beam energy decrease to ~250 MeV and the divergence increase to ~7 mrad although the beam stability was improved compared to pure helium. With 100% nitrogen, the beam divergence significantly increased to >30 mrad, and the energy dropped below 150 MeV.

The THz signal, however, shows an opposite trend. Here the signal represents the magnitude of the negative peak voltage obtained from the pyroelectric detector as shown in Fig. 2c. Contrary to the beam quality, the THz signal increased with the growing nitrogen concentration. At our laser intensities, molecular nitrogen provides 5 times more free electrons compared to helium. Thus more electrons were produced in nitrogen compared to helium, but fewer electrons were trapped into the plasma buckets for wakefield acceleration as shown in Fig. 2b. This suggests that it is not the high-energy (>150 MeV) electron bunch that contributes to THz generation. We note that the fast positive voltage spike appearing at *t* = 0 is possibly due to an electromagnetic pulse (EMP) produced from laser-produced plasma and subsequently coupled into the detector cable. The signal was the strongest with pure nitrogen, but the spike voltage was not directly correlated with the THz signal.

**Fig. 3:**
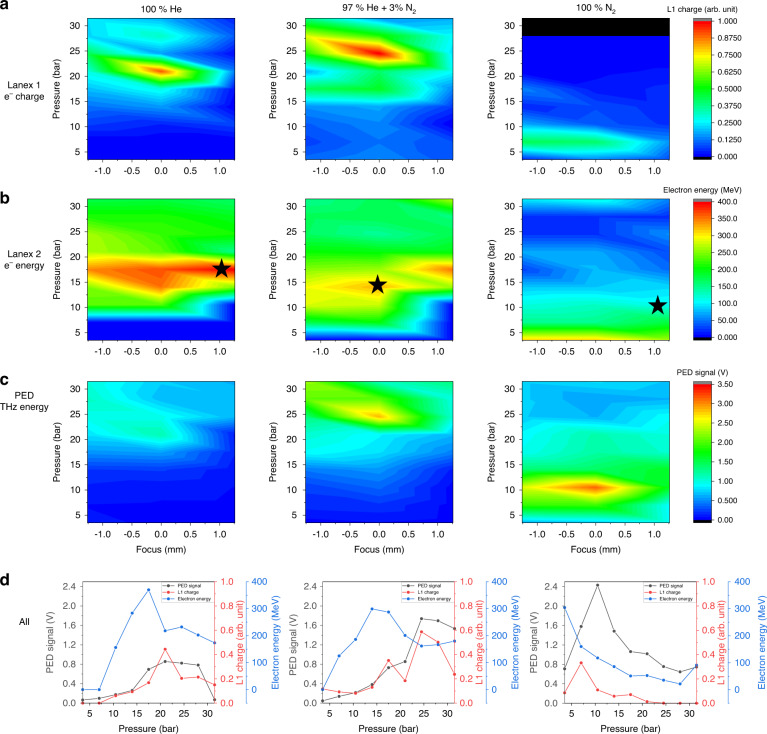
Electron and THz properties. **a** Electron charge obtained from Lanex 1, **b** electron peak energy from Lanex 2, and **c** THz pyroelectric detector (PED) signal, all measured with varying the focal position (*x*-axis) and gas backing pressure (*y*-axis) for 100% helium, 97% helium mixed with 3% nitrogen, and 100% nitrogen gas targets. **d** Electron charge (red line), peak energy (blue line), and THz signal (black line) as a function of pressure. For (**a**–**c**), a 2-D contour color fill method was adopted, with real measurements made at three focal positions (−1.26, 0, and 1.26 mm) and nine pressures from 3.5 to 31.5 bar with an increment of 3.5 bar. The black stars represent the data selected for Fig. 2. The black stripe in (**a**) for 100% nitrogen indicates that no data was taken due to strong EMPs

To investigate the origin of THz radiation, the THz signal was measured simultaneously with the electron beam energy and charge at various gas densities and laser focal positions. Plotted in Fig. 3 are the relative (a) electron bunch charge observed on Lanex 1 (collected within ~2°), (b) peak electron energy detected by Lanex 2, and (c) THz signal, all for the three gas species. In most cases, the signals were strong when the laser was focused near the center of the gas target. As shown in (b), pure helium yields the highest electron energy among all gases. The energy peaks at 17.5 bar with estimated gas and electron densities of 2 × 10^18 ^cm^−3^ and 4 × 10^18 ^cm^−3^, respectively. The corresponding plasma wavelength *λ*_p_ = 16 μm is nearly twice the laser pulse length, *cτ* = 8.1 μm, where *τ* = 27 fs is the laser pulse duration, satisfying an optimal condition for LWFA^21^.

For 97% helium and 3% nitrogen, the peak electron energy occurs around 15 bar, slightly lower than 17.5 bar due to the 3% nitrogen mixture, but with the same electron density of *N*_*e*_ = 4 × 10^18 ^cm^−3^ as in pure helium. Interestingly, the THz peak signal appears at much higher pressures (~25 bar) with *N*_*e*_ = 6.4 × 10^18 ^cm^−3^, where the Lanex 1 signal also peaks. By contrast, the Lanex 2 signal (high-energy charge, not shown in Fig. 3) enhances around 15 bar. This suggests that the THz signal is more correlated with the electron bunch charge, not the energy. This trend is also seen in pure nitrogen which gives the highest THz signal at 10 bar with *N*_*e*_ = 1.1 × 10^19 ^cm^−3^. This is more or less consistent with the bunch charge maximally observed around 7.5 bar on Lanex 1. The electron energy appears to peak below 3.5 bar, as expected from the optimal condition for LWFA in nitrogen.

Commonly observed in all gas species is that the electron energy gradually grows with increasing pressure (or electron density) and peaks at its optimal electron density and then continuously drops as shown in Fig. 3d. At 1.6−3 times the optimal density for LWFA, the bunch charge peaks together with the THz signal, and then both decrease. This indicates that the bunch charge is more critical than the energy in THz generation. More interestingly, pure nitrogen produces the strongest THz signal, but its charge signal observed on Lanex 1 is lower than that of 3% nitrogen mixture. This implies that many low-energy large-divergence electrons, not detected by Lanex 1, also play a big role in THz generation.

### THz properties

**Fig. 4:**
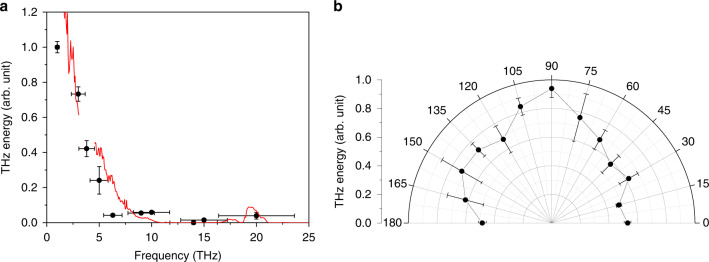
THz spectrum and polarization. **a** Discrete THz spectrum characterized with bandpass filters for the pure nitrogen gas target. The horizontal error bars represent the spectral width (FWHM) of the filter’s transmission band. The red line represents an overall transmission curve of all filters placed in the beam path. **b** THz polarization characterized with a wire grid polarizer. The laser is linearly polarized along the horizontal axis (0 or 180°)

The emitted THz radiation was characterized by its spectrum, energy, and polarization. The spectrum of the collected THz radiation was measured with metal-mesh bandpass filters. For each filter, 3−5 shots were collected and averaged, and then the spectral power at each filter frequency (circles) was obtained by normalizing the detected pyroelectric signal with the peak transmission value of the filter. The resulting THz spectrum is shown in Fig. 4a in scatters. It extends beyond 10 THz, but most of the energy is concentrated below 5 THz. This is because the spectrum was strongly affected by the filters used in the beam path. The red line in Fig. 4a represents the overall transmission curve allowed by all filters (IR filter, mylar window, Si beamsplitter, and lowpass filter), and it closely follows the measured spectrum. This implies that the real spectrum can be much broader than the measured one in Fig. 4a.

The responsivity of the pyroelectric detector was characterized to be 0.22 μJV^−1^ at 800 nm in a single-shot mode and converted to an averaged value of 0.35 μJV^−1^ at 1−10 THz by using calibration data provided by the vendor. See “Materials and methods” section (also Fig. S3) for the energy and spectral calibration of the detector. The highest THz signal was 5.9 V, obtained with nitrogen at ~10 bar with an input laser energy of 2.7 J. The corresponding THz energy is 4 mJ, estimated right after the holed OAP, with the total transmission (0.055%) of all filters considered. This gives a conversion efficiency of 0.15% or 0.5%, depending on whether the input laser energy is taken from the whole beam or within the first Airy disk of the focused laser beam (see “Materials and methods” section). The real THz energy and efficiency could be much higher as the detection bandwidth was strongly constrained by the filter transmission, mostly due to the mylar window and lowpass filter as shown in Fig. S4.

The polarization of the emitted THz radiation was characterized by using a wire grid polarizer (MICROTECH G30x10-S) with the pyroelectric detector. At every 15° of rotation of the polarizer, 3−5 pyroelectric signals were collected and averaged. The result is shown in Fig. 4b. The measured polarization distribution is isotropic, in agreement with radial polarization expected from both longitudinal and radial acceleration of electrons. There is some noticeable enhancement along the vertical polarization direction, which might have been caused by the holed OAP mirror that was slightly off-centered such that the radially polarized THz radiation acquired asymmetric polarization upon reflection. The extinction ratio of the polarizer was 30:1 when tested with another wire grid polarizer (MICROTECH G50x20-S) under the same radiation condition.

### Model and simulation

To understand the origin of high-energy THz generation from LWFA, we first estimate the maximum possible THz energy produced by CTR under our experimental conditions. With an assumption of the central radiation wavelength of 2*cτ* = 16 μm, the total energy emitted over all angles by CTR from a 15-pC, 200-MeV electron bunch is 3 μJ (see “Materials and methods” section). This is about 3 orders of magnitude lower than 4-mJ observed in our experiment. This is because the bunch charge is simply too low to produce multi-mJ THz energy. CTR can be also produced by high-charge (~nC) low-energy electron bunches produced continuously with the laser propagation, not injected into the bubble for high-energy acceleration. However, those electrons will spread in space and time with propagation, not fully satisfying the CTR condition when they arrive at the plasma-vacuum boundary. Those electrons have an average divergence angle of several degrees in our experimental condition. A propagation distance over 4 mm would make the bunch diameter more than 700 μm on average at the plasma-vacuum boundary, which far exceeds the expected THz wavelength.

Alternatively, we consider coherent radiation by copious low-energy electrons suddenly accelerated by the ponderomotive force and plasma wakefields on the time scale of the laser pulse duration and plasma period. Such radiation can be coherent and scale with the charge squared because the source dimensions are comparable to the laser focal volume. Moreover, the source extends over the entire plasma length, and the radiation emitted continuously along the propagation direction can constructively interfere (phased-matched) to produce high-energy coherent THz pulse in the far field. This type of radiation can yield THz energy as high as 14 mJ at <40 THz under our experimental conditions as discussed in “Materials and methods” section.

**Fig. 5:**
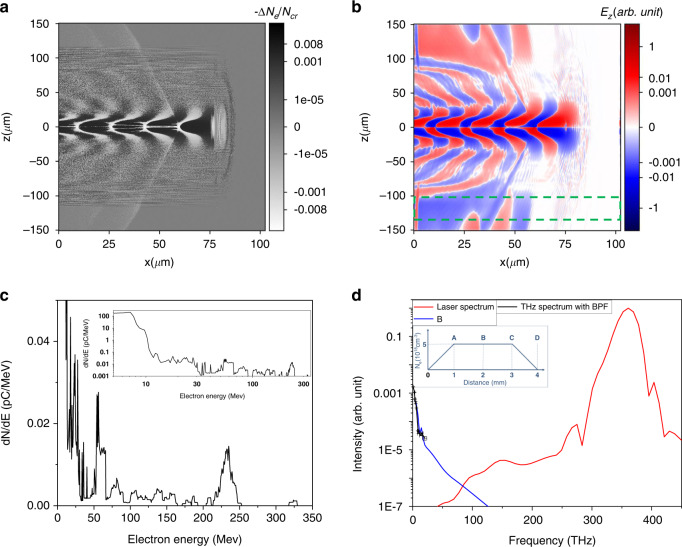
PIC simulation results for pure helium obtained with ***ɑ***_**0**_ = 1.9 and plasma density of 5 × 10^**18** ^cm^**−3**^. **a** Plasma electron density modulation (−Δ*N*_*e*_) normalized by *N*_*c*_ and **b** *E*_*z*_ distribution after the laser propagating over 3 mm in He. **c** Electron energy spectra. Inset: y-axis on a log scale. **d** Fourier spectrum (blue line) of *E*_*z*_(*x*) summed over *z* = 100−130 μm calculated at Point B in the trapezoidal density profile (inset), coplotted with the laser spectrum (red line) and measured THz spectrum (black line) retrieved from Fig. 4a with the detector’s responsivity and filter/window transmission curves included (see Figs. S3b and 4a)

To have a better understanding of THz generation in LWFA, particle-in-cell (PIC) simulations were conducted. In the simulations, the gas density profile was assumed to be trapezoidal along the laser propagation direction (+*x*-axis) as shown in the inset to Fig. 5d, with a plateau density of *N*_*e*_ = 5 × 10^18 ^cm^−3^ when fully ionized. A 27-fs, 800-nm laser pulse with linear polarization (*y*-axis) is vacuum-focused at *x* = 1 mm (Point A in the inset) with *a*_0_ = 1.9. Figure 5a shows an electron density modulation ($- \Delta N_e$) normalized to the critical density *N*_*c*_ = 1.75 × 10^21 ^cm^−3^ at 800 nm when the pulse reaches at *x* = 3 mm (Point C). The corresponding electric field distribution (*E*_*z*_), perpendicular to the laser polarization, is shown in Fig. 5b on a symlog scale as in Fig. 5a. It shows highly nonlinear wakefield structures within z = ±30 μm, as well as traveling THz-frequency electromagnetic waves outside. The same waves appear on the *xy* plane, indicating they are radially polarized. They are related to the electron spectrum in Fig. 5c which shows a 200–250 MeV electron bunch of <1 pC charge, together with numerous (~nC) low-energy (<10 MeV) electrons. These low-energy electrons are initially created by the laser field and then suddenly pushed away (or accelerated) by the laser ponderomotive force. Upon returning due to the electrostatic force, they are strongly scattered (or accelerated) at the back of the plasma bucket where the electron charge density highly peaks. Many electrons, not being injected into the plasma bucket for steady acceleration, undergo this sudden acceleration. These scattered electrons are observed in Fig. 5a as tilted wakes (or streaks), propagating at ~10° to the forward direction. This scattering occurs on the time scale of the plasma period and within a fraction of the bubble volume, thus producing coherent THz radiation in the far field. The Fourier spectra of the THz waves inside the green dashed box are plotted in Fig. 5d. Here the spectral intensity considers the local THz waves within a cylindrical shell with 0 ≤ *x* ≤ 100 μm and 100 μm ≤ |*z* | ≤ 130 μm, not covering all THz waves propagating beyond the moving frame. Nonetheless, the THz intensity is strong, about ~1500 times lower than that of the laser (red line) in Fig. 5d.

## Discussion

The measured THz energy was as high as 4 mJ at 1-10 THz, but the energy was strongly cut by the filters used in the beam path, mostly by the mylar window and the lowpass filter. With a better choice of THz optics, a few times more THz energy is expected to be detected. In addition, the effective laser energy used to drive LWFA was <30% of the focused energy. With more careful DM control, the energy concentration within the first Airy disk can be enhanced further beyond 50%. This will increase the output THz energy even further.

A better THz vacuum window will also allow us to correctly characterize the radiation spectrum. The mylar window was selected to pre-align the THz beam path more conveniently with visible light. However, it turns out to be quite absorptive at 5−15 THz (see Fig. S5c) and strongly attenuated THz radiation. Another choice for the vacuum window is high resistivity float zone (HRFZ) silicon (Si). It provides a nearly flat transmission (50%) value over a broad range, but it is also quite lossy at 15−30 THz (see Fig. S5b). As both windows provide a significant transmission loss, it is advisable to use this source inside a vacuum chamber when conducting any strong THz-driven experiments.

In conclusion, we have observed multi-mJ THz emission from 100-TW-laser-driven LWFA with an energy conversion efficiency of 0.15%. The emitted THz radiation is radially polarized and broadband, possibly extending beyond 10 THz. The correlation between the electron beam properties (energy and charge) and THz output energy shows that high-energy (>150 MeV) electrons do not necessarily yield high-power terahertz radiation. Instead, low-energy (<MeV) but high-charge electrons can produce much stronger terahertz radiation. To explain this interesting result together with multi-mJ THz generation, we have proposed a coherent radiation model, in which the electrons accelerated by the laser ponderomotive force and subsequent plasma wakefields radiate broadband emission continuously along the laser propagation direction, ultimately resulting in phase-matched conical THz radiation in the far field. This model, however, needs to be verified or examined by more follow-up experiments and analytic/numerical studies in order to have a full understanding of THz generation in LWFA, as well as to optimize the source for future high-power THz applications.

## Materials and methods

### Laser system and diagnostics

The experiment was based on a 150-TW Ti:sapphire laser capable of producing >25-fs, 4-J pulses (after compression) at a repetition rate of 5 Hz, housed at the Center for Relativistic Laser Science (CoReLS), Institute for Basic Science (IBS), South Korea^28^. The laser was previously used to demonstrate LWFA for electron beam energy enhancement with plasma density shaping^32^, optical shaping of wakefields^33^, and nanoparticle-assisted electron injection^34,35^. In this experiment, the laser was operated in a single-shot mode to avoid thermal degradation of the laser’s compressor gratings under high-average-power operation. The laser pulse was p-polarized (parallel to the optical table), and its spectrum was centered at 800 nm with a 60-nm bandwidth in FWHM. Starting from the pulse compressor, the laser beam was transported in vacuum (<10^−4^ mbar) with a beam diameter of 60 mm. The laser pulse duration was minimized by characterizing and controlling group delay dispersion (GDD) and higher order dispersion with a combination of an acousto-optic programmable dispersive filter (FASTLITE Dazzler) and an ultrafast laser pulse characterizer (APE FC Spider). The laser energy at the target was controlled by rotating a half-wavelength (λ/2) waveplate located before the compressor.

The pulse wavefront was characterized and corrected by an adaptive optical system consisting of a wavefront sensor (PHASIC SID4-GE) and a deformable mirror (DM)(AKA OPTICS DM2-60-32). The DM was essential to correct the wavefront after energy amplification and to pre-compensate the wavefront as the laser beam was clipped at the center when reflected by a 45° mirror with a hole (20-mm diameter) in the target chamber (see Fig. S1). The DM was also used to move the vacuum focal point by simply applying a constant defocus voltage to all mirror segments. This method was adopted to take the data shown in Fig. 3. At the target chamber, the laser pulse was focused by a 76-mm-diameter concave mirror with the focal length of 1.5 m onto a gas target. The beam profile at the focus was characterized by a focal spot monitor (FSM) consisting of a ×10 microscope objective (MITUTOYO APO NIR 10x) and a 3840 × 2764 camera (EPIX SV10M6). After DM optimization, the focused beam size was 22 μm in FWHM (see Fig. S2a, b), with <30% energy concentrated within the Airy disk. The intensity profile is close to a Gaussian shape with the beam waist *w*_0_ of 19 μm. With 27-fs and 0.8-J (within the Airy disk) pulses at the target, the peak intensity was 5.2 × 10^18^ W/cm^2^ (*a*_0_ = 1.6).

### Gas target and electron beam characterization

The gas jet was produced by a solenoid valve (PARKER Series 9) through a cylindrical nozzle (4 mm inner diameter) in pulsed mode with an opening time of 10 ms. The gas density profile was characterized by using a wavefront sensor (PHASICS SID4-HR) that measures the phase shift imposed upon a probe laser beam propagating through the gas. At backing pressures of 7−28 bar, the gas densities were measured to be 0.8−3.0 × 10^18 ^cm^−3^ at the laser beam height of 2.0 mm from the nozzle tip. The density profile was nearly flat over 2 mm with 1-mm up and down ramps on the sides. The laser-irradiated plasma was monitored by two 14-bit, 1392 × 1040 cameras (PCO pco.pixelfly) for the side and top views (see Fig. S2c, d).

The electron beam generated by LWFA was characterized with three scintillating Lanex (KODAK) screens. The electron beam profile, pointing, divergence, and relative charge was detected by Lanex 1, placed at 320 mm from the gas jet and tilted at 45° to the beamline. The electron beam energy was measured with an electron spectrometer consisting of a 1-T dipole magnet that has an opening of 205 mm (L) × 70 mm (W) × 8 mm (H), together with Lanex 2 and 3 screens. Lanex 2 and 3 were placed just after and 536 mm away from the end of the magnet to detect electron energies at 120−350 MeV and 150−400 MeV, respectively. Each Lanex screen was monitored by a 16-bit, 2560 × 2160, sCMOS camera (PCO pco.edge 5.5) in real time. Due to radiation safety concerns, all experimental data were taken remotely in a control room. The electron charge was calibrated by using an imaging plate (IP)(FUJIFILM BAS-MS), which was placed on top of the Lanex 3 screen and exposed to 3 cumulative shots. Later, it was read by an IP scanner (FUJIFILM BAS5000) to determine the total charge, considering the photostimulated luminescence (PSL) of the IP, energy dependence of PSL, and time elapse^36–38^. The charge at >150 MeV was estimated to be in the range of <15 pC.

### THz detector and optics characterization

For accurate measurements of THz energy, the pyroelectric detector (GENTEC THz5I-BL-BNC) was calibrated by using a femtosecond Ti:sapphire laser capable of providing 800-nm, 30-fs, 1-mJ pulses at 1 Hz. The energy of the laser pulse, after attenuated by a set of neutral density filters, was directly measured with an energy meter (OPHIR PHOTONICS PE10-C). The energy-to-voltage response was linear over a wide range of the pyroelectric voltage (see Fig. S3a). The responsivity of the detector, obtained from least squares fitting of the slope, was 0.220 ± 0.008 μJV^−1^. This was cross-checked by using a CW laser diode operating at 633 nm. The laser beam was optically chopped by a low-duty-cycle (<0.5%) wheel at various chopping frequencies. The responsivity obtained at the DC frequency limit was 0.210 ± 0.008 μJV^−1^, in good agreement with the first method. The responsivity at THz frequencies was obtained by matching the spectral correction curve provided by the vendor at 800 nm (see Fig. S3b). In general, the pyroelectric detector yields a relatively flat response over a wide range of optical and infrared frequencies, but it exhibits rapidly increasing responsivities at <5 THz, due to reduced energy absorption by the organic coating of the detector sensor. As a result, the detector provides responsivities of 0.24 μJV^−1^ at 10 THz, 0.30 μJV^−1^ at 5 THz, 0.35 μJV^−1^ at 3 THz, 0.35 μJV^−1^ averaged at 1−10 THz, and 0.50 μJV^−1^ averaged at 1−5 THz.

The highest pyroelectric voltage was 5.9 V, obtained with several filers and windows placed in the beam path as shown in Fig. S4. Those include two IR filters (EDMUND OPTICS) to attenuate any possible optical leakage, a 180-μm-thick mylar vacuum window, a 6-mm-thick beamsplitter (TYDEX BS-HRFZ-SI-D76.2-T6), a 10% energy attenuator (TYDEX ATS-5-50.8), a lowpass filter (TYDEX LPF23.1-47) transmitting radiation below 23.1 THz, and a 2-mm-thick Si filter (EDMUND OPTICS) placed to protect the detector. Here the transmission of each optic was measured by three different sources using (i) the emitted THz radiation itself while comparing the pyroelectric signals (3−5 shots averaged) with and without an identical optic placed in the beam path, (ii) a quantum cascade laser (QCL) operating at 3.1 THz, and (iii) Ti:sapphire laser-based THz time-domain spectroscopy (TDS) at 0.3−3.0 THz.

All transmission values measured by the three methods are tabulated in Fig. S4. Most values are consistent except for the mylar window. This discrepancy is possibly due to the spectral range. As shown in Fig. S4b, the overall transmission curve allowed by all filters and windows covers a much broader range than TDS (0.3−3.0 THz). The mylar window is expected to give a lower value (36% instead of 52%) due to its increasing absorption with frequency as shown in Fig. S5c. The transmission averaged at 0.3−10 THz, calculated from the total transmission curve shown in Fig. S4b, is 0.055%. We note that the filter spectrum shown in Fig. S4b is different from the red line in Fig. 4a because of the additional 2-mm-thick Si filter, which strongly absorbs radiation below 3 THz (see Fig. S5c).

The highest THz energy estimated right after the holed OAP is 4 mJ at 1−10 THz, obtained from the filter transmission of 0.055% and the pyroelectric responsivity of 0.35 μJV^−1^, both averaged at 1−10 THz. The transmission of all filters and windows used in the experiment was characterized at 0.1−100 THz with a combination of Fourier transform infrared (FTIR) spectroscopy (BRUKER Vertex 70 v) and THz-TDS (see Fig. S5).

### PIC simulation

In order to simulate LWFA, we have performed 3D PIC simulations using SMILEI^39^ in the cylindrical geometry. A laser pulse with a Gaussian profile (*ɑ*_0_ = 1.9, *w*_0_ = 15 μm, τ = 27 fs, and λ = 800 nm) propagates into a 4-mm pure He gas target with a plasma density of 5 × 10^18 ^cm^−3^ when fully ionized. The ionization rate is calculated with the ADK module, which is implemented in the SMILEI code. The simulation box has dimensions of 43*c*/*ω*_*p*_ × 63.2*c*/*ω*_*p*_ moving at the speed of light along the *x*-direction, where *ω*_*p*_ is the angular plasma frequency. The box is initialized with 4096 × 807 cells along the *x* and *r* directions, respectively, using 8 particles per cell. The laser pulse propagates in vacuum over 14.7*c*/*ω*_*p*_ and interacts with a gas medium with non-uniform profile (420.4*c*/*ω*_*p*_ up-ramp, 840.9*c*/*ω*_*p*_ plateau, and 420*c*/*ω*_*p*_ down-ramp).

### Model details

We first consider CTR. The transition radiation energy E_TR_, emitted by a single electron traveling with velocity $\beta = v/c$ and passing through a plasma-vacuum boundary that is treated as the interface between a perfect conductor and vacuum for simplicity, is given by^24,25,40^

1 $$\frac{{d^2{{{\mathrm{E}}}}_{{{{\mathrm{TR}}}}}}}{{d\omega d{{\Omega }}}} = \frac{{e^2}}{{\pi ^2c}}\frac{{\beta ^2\sin ^2\theta }}{{\left( {1 - \beta ^2\cos ^2\theta } \right)^2}}$$

where *ω* is the angular frequency of the radiation, Ω is the solid angle, and *θ* is the radiation angle with respect to the electron trajectory. In the highly relativistic limit *β*→1, the total radiated energy over all angles is approximated as

2 $$\frac{{d{{{\mathrm{E}}}}_{{{{\mathrm{TR}}}}}}}{{d\omega }} \approx \frac{2}{\pi }\frac{{e^2}}{c}\ln \gamma$$

where $\gamma = \left( {1 - \beta ^2} \right)^{ - 1/2}$ is the relativistic Lorentz factor. The total energy contained within the bandwidth of THz radiation, $\Delta \omega = 2\pi /\tau$, is

3 $${{{\mathrm{E}}}}_{{{{\mathrm{TR}}}},tot} = \frac{2}{\pi }\frac{{e^2}}{c}\Delta \omega \ln \gamma = 4\frac{{e^2}}{{c\tau }}\ln \gamma$$

For an electron bunch of charge *q*, the total THz energy emitted by the bunch in ideal CTR, where the bunch size is much smaller than the radiation wavelength, is obtained by replacing the electron charge *e* with the bunch (or point) charge *q*.

We now consider radiation by acceleration. The power radiated per unit solid angle by a single electron accelerating at *a* is^41^

4 $$\frac{{dP_a}}{{d\Omega }} = \frac{{e^2a^2}}{{4\pi c^3}}\frac{{{{{\mathrm{sin}}}}^2\theta }}{{\left( {1 - \beta \cos \theta } \right)^5}}$$

when the acceleration ***a*** is parallel to the velocity ***β***. The total power emitted over all angles is given by

5 $$P_{a,tot} = \frac{2}{3}\frac{{e^2}}{{c^3}}a^2\gamma ^6$$

In highly relativistic acceleration, the electron is accelerated from rest to a large fraction of the speed of light in the laser propagation direction over the rising period of the laser pulse. This gives $a \approx c/\tau$. The total energy emitted into all angles by a single electron over the laser pulse duration is then

6 $${{{\mathrm{E}}}}_{a,tot} = P_{a,tot}\tau \approx \frac{2}{3}\frac{{e^2}}{{c\tau }}\gamma ^6$$

In both CTR and radiation by acceleration, the radiated energy scales with the charge squared, yielding coherent radiation, when the bunch size is smaller than the radiation wavelength. The above equations also show that ${{{\mathrm{E}}}}_{{{{\mathrm{TR}}}},tot} \propto \ln \gamma$ and ${{{\mathrm{E}}}}_{a,tot} \propto \gamma ^6$, indicating that the radiation by acceleration is dominant over CTR for *γ* > 1.

A propagation effect should be considered in determining the total radiation energy emitted in the forward direction. The THz waves, emitted from every point along the laser-produced plasma of length *l*, interfere in the far field, ultimately producing a donut-shaped (conical) radiation pattern. Note that $dP_a/d{{\Omega }} = 0$ at *θ* = 0°. The boundary angle *θ*_2_ of the conical profile is estimated by setting the waves emitted from the centers of the front-half and rear-half of the plasma to interfere destructively with a path length difference equal to the half of the expected radiation wavelength $\lambda _{{{{\mathrm{rad}}}}}$. This is expressed as $l\left( {c/v_g} \right)/2 - l\cos \theta _2/2 = \lambda _{{{{\mathrm{rad}}}}}/2$, where $v_g \approx c\sqrt {1 - N_e/N_c}$ is the laser group velocity in the plasma of density *N*_*e*_, and *N*_*c*_ is the critical density. With $v_g \approx c$, $\lambda _{{{{\mathrm{rad}}}}} = 2c\tau$ = 16 μm, and $l = 2z_R$ ≈ 2.8 mm, where $z_R = \pi w_0^2/\lambda$ is the Rayleigh length at *λ* = 800 nm, we get a half boundary angle of *θ* = 6.2°. Then the THz energy emitted between *θ*_1_ = 3.4° and *θ*_2_ = 6.2° by a single electron is calculated as

7 $${{{\mathrm{E}}}}_a = P_a\tau = \tau \frac{{e^2a^2}}{{2c^3}}\mathop {\int }\nolimits_{{\it{\theta }}_1}^{{\it{\theta }}_2} \frac{{{{{\mathrm{sin}}}}^2\theta }}{{\left( {1 - \beta \cos \theta } \right)^5}}\sin \theta d\theta$$

where *β* ≈ 0.75 from *a*_0_ = 1.6 and $\gamma = \sqrt {1 + a_0^2/2}$ ≈ 1.5 in our experiment. The average acceleration during *τ* is approximated as $a \approx v_d/\tau$, where $v_d = a_0^2c/\left( {4 + a_0^2} \right)$ ≈ 0.4*c* is the longitudinal drift velocity by the ponderomotive force for *a*_0_ = 1.6. From these values, we get *E*_*a*_ = 4.8 × 10^−26 ^J. To be coherent radiation, the THz waves emitted from the plasma source with the transverse size *D* must constructively interfere: $D\sin \theta \ll \lambda _{{{{\mathrm{rad}}}}}$, where *D* ≈ 2*w*_0_ is the plasma diameter. This gives *θ* $\ll$ 25°, which satisfies *θ*_2_ = 6.2° $\ll$ 25°.

Finally, the total THz radiation energy, emitted by *N* number of electrons in each plasma bucket and summed over the plasma length *l*, is given by ${{{\mathrm{E}}}}_{a,tot} = {{{\mathrm{E}}}}_aN^2\left[ {l/\left( {c\tau } \right)} \right]$, where $N = N_e\left( {\pi w_0^2\tau /2} \right)$ is the number of electrons created and initially accelerated by the ponderomotive force within the front half of the laser focal volume. From *N*_*e*_ = 6.4 × 10^18 ^cm^−3^, *w*_0_ = 19 μm, *τ* = 27 fs, and *l* = 2.8 mm in our experiment, we get *N* ≈ 3 × 10^10^ (~5 nC) and ${{{\mathrm{E}}}}_{a,tot}$ ≈ 14 mJ. We note that this calculation considers the radiation driven by the laser ponderomotive force only, but a similar treatment can be applied to calculate the radiation by the plasma wakefields at the back of the plasma bubble.

In principle, the acceleration in the transverse direction can produce coherent THz radiation, but the local field is radially polarized under a symmetric ponderomotive force, which cancels out coherent radiation in the forward direction (*θ* = 0). Coherent radiation may be considered at an off-axis angle, similar to the longitudinal acceleration case. However, the expected radiation angle of 25°, obtained from $D\sin \theta = \lambda _{{{{\mathrm{rad}}}}}$ for $\lambda _{{{{\mathrm{rad}}}}} = 16$ μm, is much greater than the longitudinal phase matching angle of 6.2°, not to mention the OAP boundary angle of 14°. Because of this, we expect almost no coherent radiation by the transverse acceleration. Consequently, coherent radiation in the far field is mostly dominated by the longitudinal acceleration and deceleration in the laser propagation direction.

## Supplementary information

Supplementary Material
