# Supplementary material for: Multi-millijoule terahertz emission from laser-wakefield-accelerated electrons
Source: Light Sci Appl. 2023 Feb 6;12:37. doi: 10.1038/s41377-022-01068-0 (PMC9899771; doi:10.1038/s41377-022-01068-0)
Supplement: Supplementary file 1 — Supplementary Material [file 41377_2022_1068_MOESM1_ESM.docx]

**Supplementary Information for**

**Multi-millijoule terahertz emission from laser-wakefield-accelerated electrons**

Taegyu Pak^1,2^, Mohammad Rezaei-Pandari^1,3^, Sang Beom Kim^1,2^, Geonwoo Lee^1,2^, Dae Hee Wi^1,2^, Calin Ioan Hojbota^1^, Mohammad Mirzaie^1^, Hyeongmun Kim^4^, Jae Hee Sung^1,4^, Seong Ku Lee^1,4^, Chul Kang^4^, and Ki-Yong Kim^1,2,5,6^

^1^Center for Relativistic Laser Science, Institute for Basic Science, Gwangju 61005, Korea

^2^Department of Physics and Photon Science, Gwangju Institute of Science and Technology (GIST), Gwangju 61005, Korea

^3^Laser and Plasma Research Institute, Shahid Beheshti University, Tehran, Iran

^4^Advanced Photonics Research Institute, GIST, Gwangju 61005, Korea

^5^Institute for Research in Electronics and Applied Physics and Department of Physics, University of Maryland, College Park, Maryland 20742, USA

^6^[kykim@umd.edu](mailto:kykim@umd.edu)


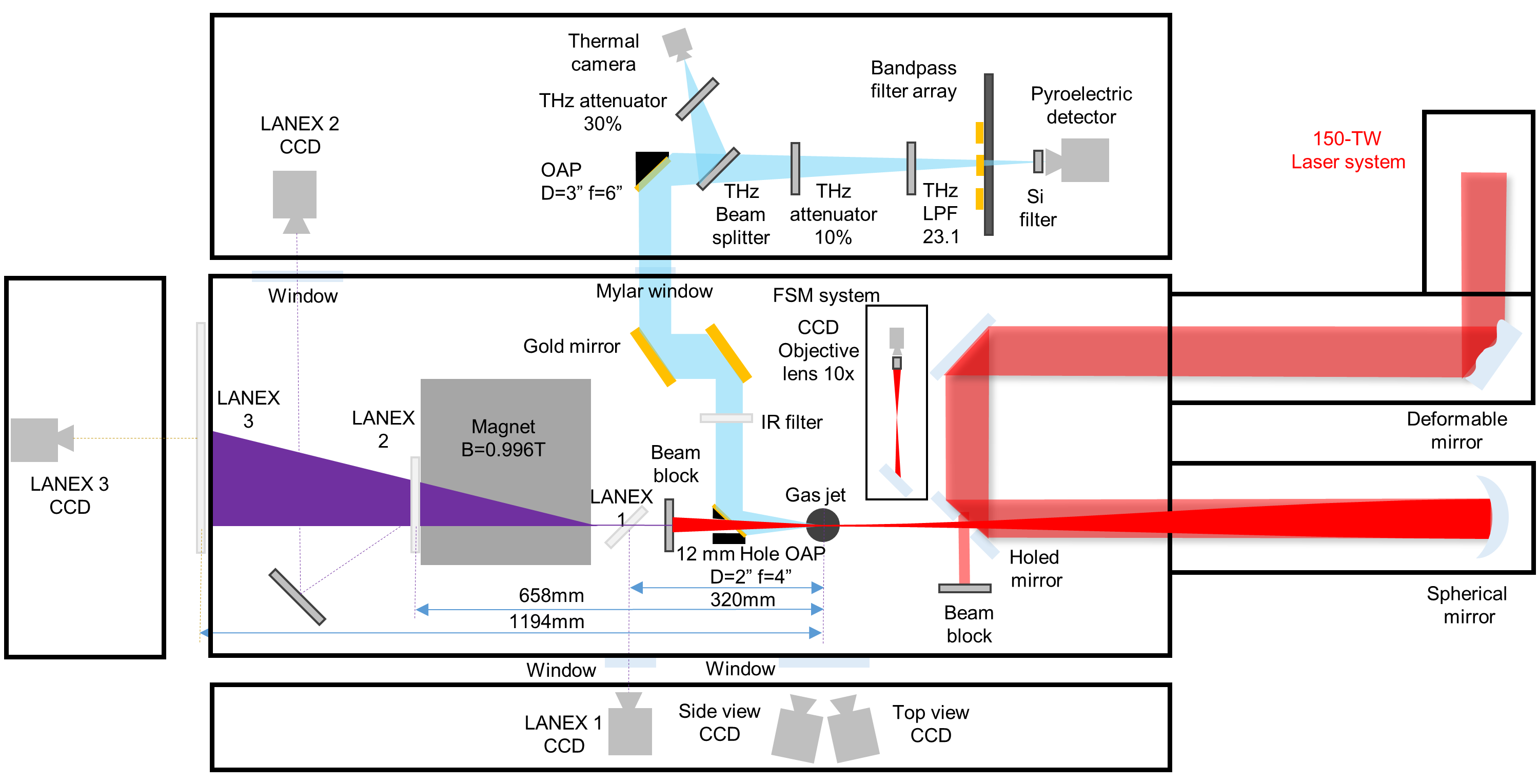


Fig. S1. Experimental layout.


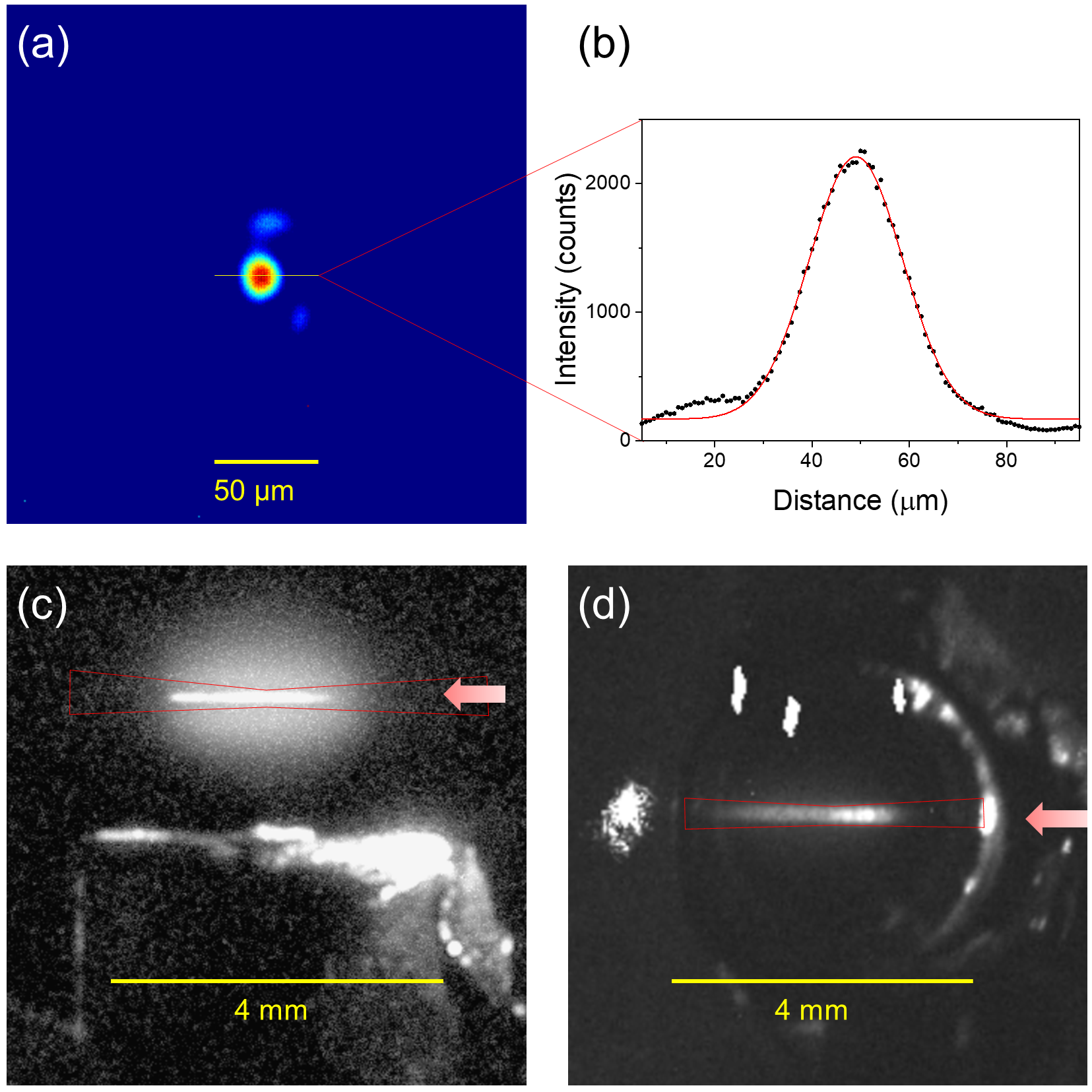


Fig. S2. (a) Focused laser beam profile after optimized with DM control. (b) Horizontal beam profile lineout. (c) Side and (d) top views of laser-produced plasma from He (97%) mixed with N_2_ (3%). In (b), the spot size is 22 μm (FWHM) with <30% of energy contained with the Airy disk, yielding a peak intensity of 5.2 × 10^18^ W/cm^2^ with *a*_0_ = 1.6.


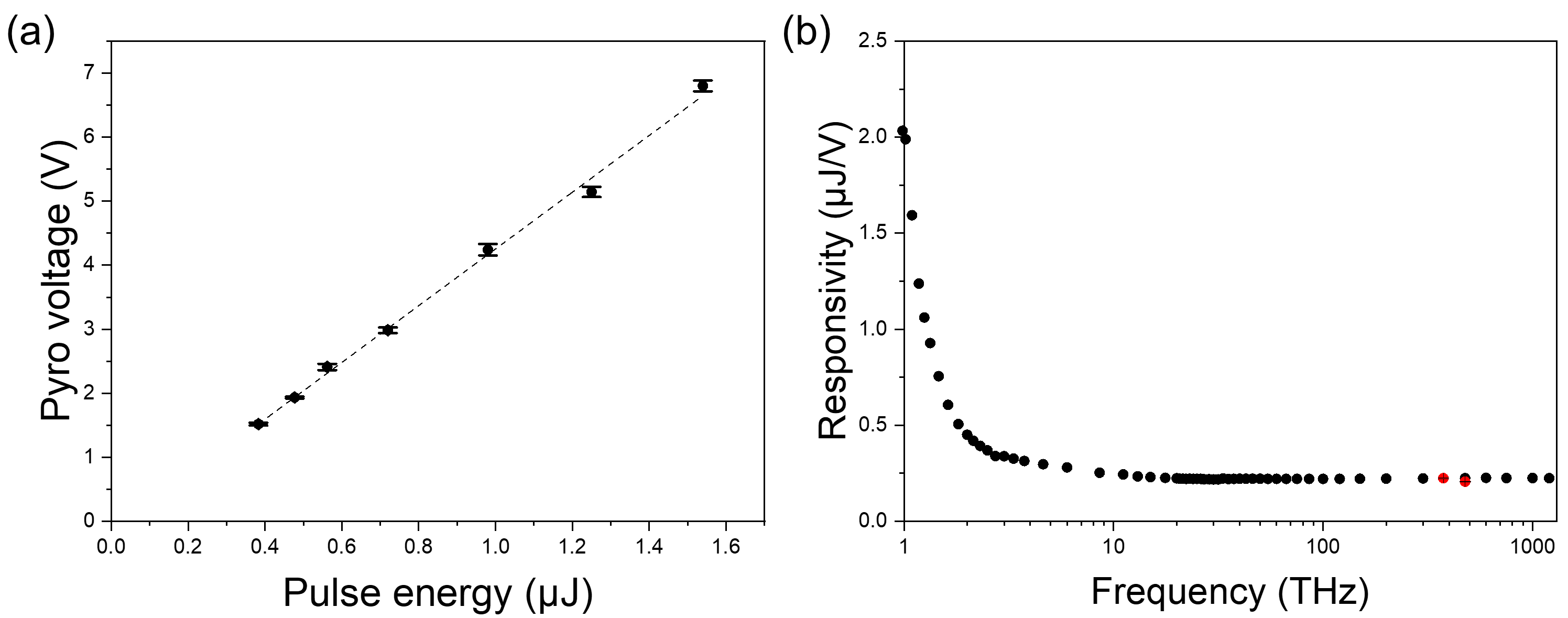


Fig. S3. (a) Energy and (b) spectral calibration of the pyroelectric detector (GENTEC THz5I-BL-BNC). The slope in (a) provides the responsivity of the detector, 0.220 ± 0.008 μJV^-1^, measured with femtosecond laser pulses at 800 nm. In (b), the spectral correction curve provided by the vendor is matched at 800 nm and converted into μJV^-1^. It provides 0.24 μJV^-1^ at 10 THz, 0.30 μJV^-1^ at 5 THz, 0.50 μJV^-1^ averaged at 1−5 THz, and 0.35 μJV^-1^ averaged at 1−10 THz.


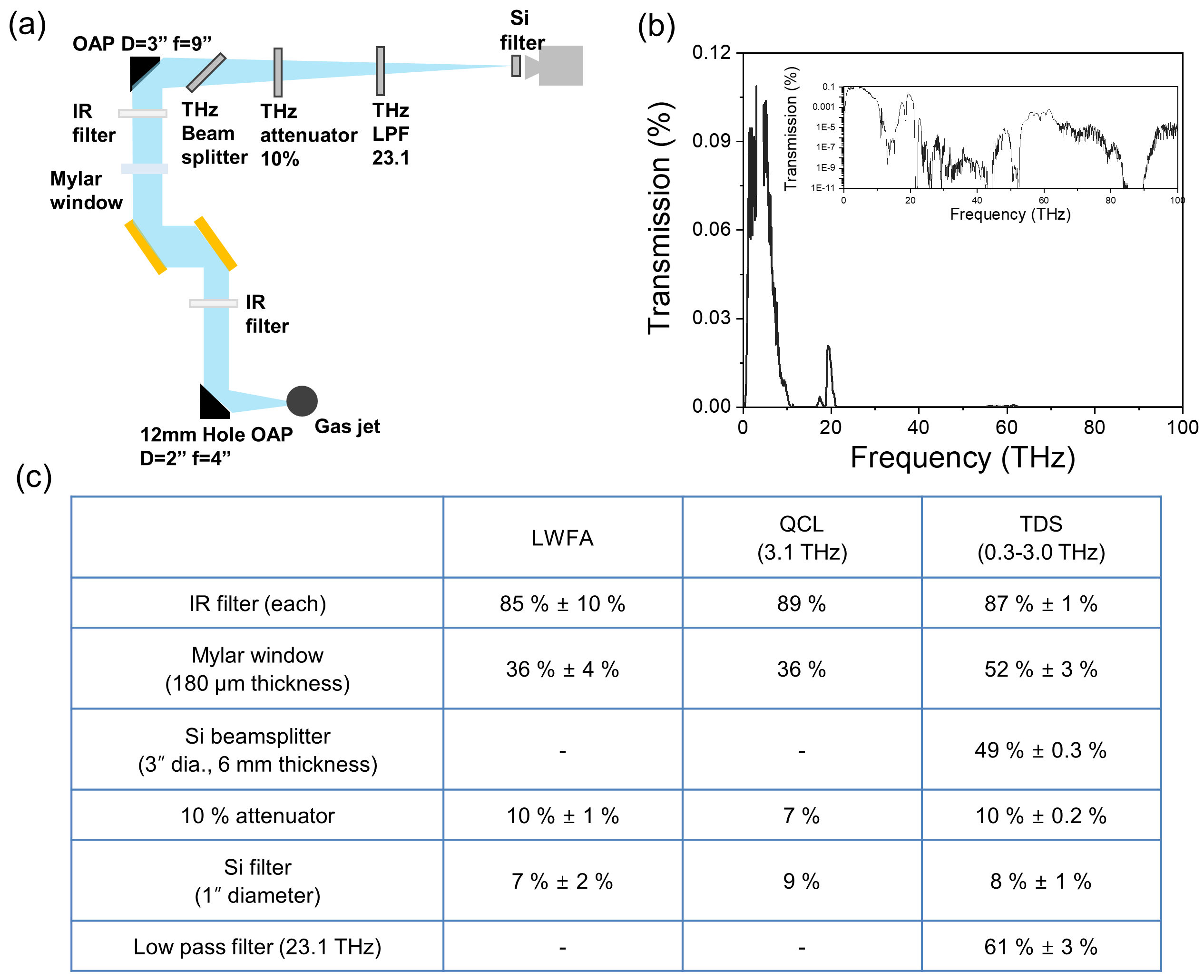


Fig. S4. (a) Filters and windows inserted in the beam path when the strongest THz signal was observed with the pyroelectric detector. (b) Overall transmission curve allowed by all filters and windows used in (a). (c) Table: THz transmission values measured by using the emitted radiation (LWFA in N_2_), a 3.1-THz QCL laser, and THz-TDS.


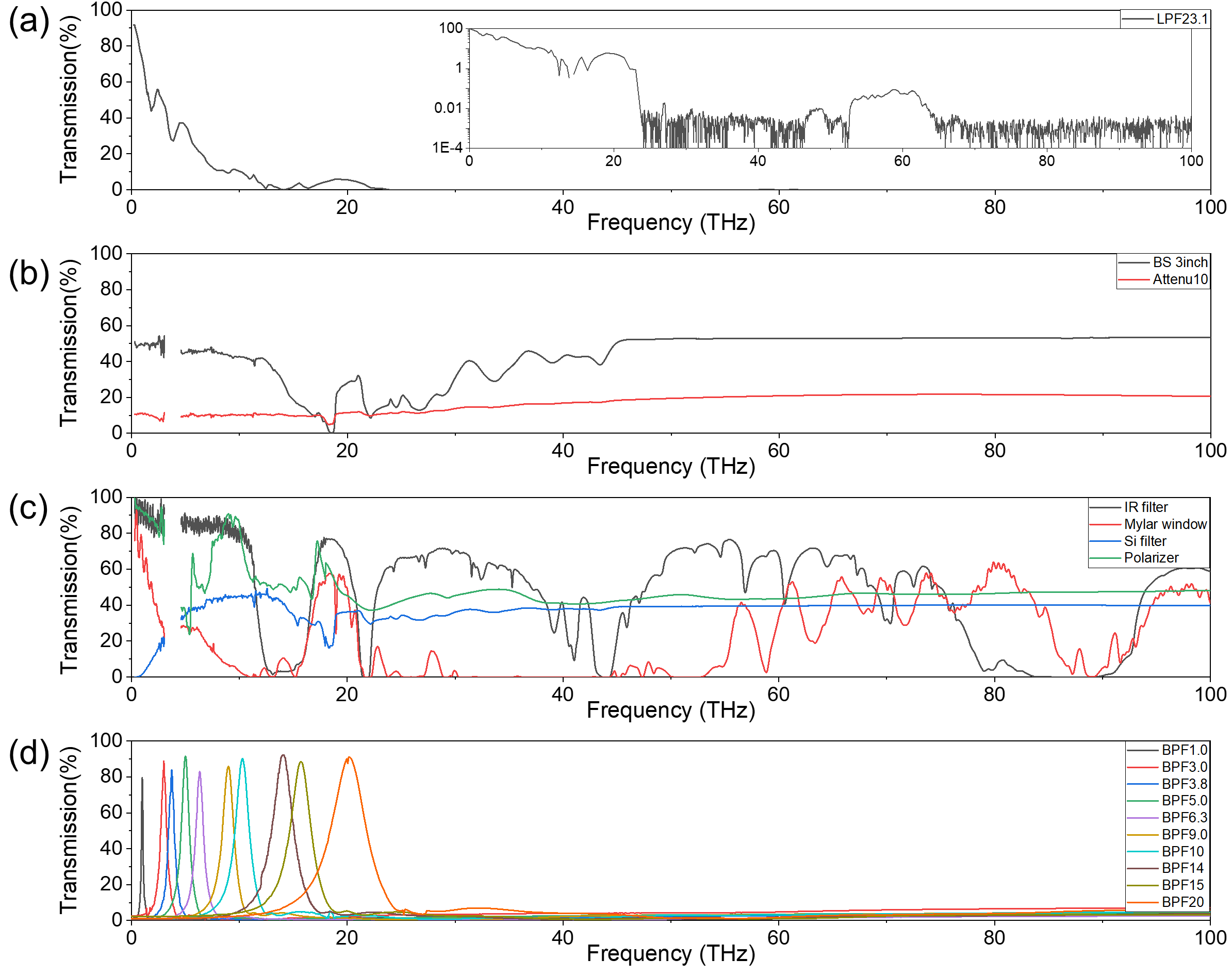


Fig. S5. Filter and window transmission curves at 0.1−100 THz frequencies, characterized by a combination of Fourier transform infrared (FTIR) spectroscopy (BRUKER Vertex 70v), Ti:sapphire laser-based THz-TDS, and/or data provided by the vendor. (a) Lowpass THz filters (TYDEX LPF23.1). Inset: y-axis on a log scale. (b) THz attenuators (TYDEX, 10% transmission) and 3”-diameter, 6-mm-thick Si beamsplitter (TYDEX BS-HRFZ-SI-D76.2-T6). (c) IR material (EDMUND OPTICS), mylar window (180-μm thickness), Si window (EDMUND OPTICS, 2-mm thickness), and THz polarizer (MICROTECH G30x10-S). (d) THz bandpass filters (THORLABS).
